# A qualitative exploration of cAregiver experienCes Of conseRvatively maNaged kidney failure: the ACORN study

**DOI:** 10.1186/s12882-025-04209-w

**Published:** 2025-07-01

**Authors:** Claire Carswell, T. Forbes, A. Wilson, G. Laurente, M. Yaqoob, P. Gilbert, S. Bolton, A. Burns, C. Moran, S. O’Neill, J. McGarry, N. O’Hare, K. McGuigan, C. McKeaveney, C. McVeigh, J. Reid, S. Rej, I. Walsh, H. Noble

**Affiliations:** 1 Department of Health Sciences, University of York, York, UK; 2 School of Nursing and Midwifery, Queen’s University Belfast, Belfast, UK; 3 Barts Health NHS Trust, London, UK; 4 Northern Ireland Kidney Patient Association, Belfast, UK; 5 Northern Health and Social Care Trust, Antrim, UK; 6 Royal Free London NHS Foundation Trust, London, UK; 7 Western Health and Social Care Trust, Londonderry, UK; 8 Belfast Health and Social Care Trust, Belfast, UK; 9 Department of Psychiatry, Lady Davis Institute, Jewish General Hospital, McGill University, Montréal, Canada; 10 School of Medicine, Dentistry and Biomedical Sciences, Queen’s University Belfast, Belfast, UK

**Keywords:** Kidney disease, Conservative management, Caregivers, Qualitative, Health & well-being

## Abstract

**Introduction:**

Patients with kidney failure who are older, frail and have multiple conditions can choose not to receive dialysis and instead receive conservative management, which focuses on symptom management and maximising quality of life. Many of these patients rely on support from informal caregivers, such as family and friends. However, the experiences of informal caregivers of people who choose conservative management, particularly towards the end of life, are generally unknown.

**Aim:**

To explore the experiences of informal caregivers of people receiving CM for kidney failure alongside healthcare professionals who provide treatment and care to people receiving CM to identify and understand the unmet needs of informal caregivers.

**Methods:**

Informal caregivers of patients receiving conservative management (*n* = 38) were recruited from five sites, two in England and three in Northern Ireland. Semi-structured interviews focused on the experiences of the caring role were conducted with informal caregivers and thematically analysed. Focus groups and one semi-structured interview were conducted at two sites (one in Northern Ireland, one in England) with healthcare professionals who had experience caring for people receiving conservative management (*n* = 15). The focus groups explored their experience of supporting informal caregivers and their perspectives on the needs of informal caregivers. These were thematically analysed, and the analysis from the two data sets (informal caregivers and healthcare professionals) was integrated.

**Results:**

Three themes and nine subthemes were synthesised from the data. These included: Defining the role of ‘carer’, which captured perspectives on the caring role and motivations for caring; keeping the end in mind, which described attitudes and awareness of disease progression and death; and balancing the burden, which encapsulated the ways informal caregivers managed the burden of caring.

**Conclusion:**

Informal caregivers had multifaceted experiences and felt well-supported by renal healthcare teams. Cultural factors strongly influence caregivers’ experiences of the role, while communication and the provision of information were identified as the key needs of caregivers.

**Clinical Trial Number:**

Not applicable.

**Supplementary Information:**

The online version contains supplementary material available at 10.1186/s12882-025-04209-w.

## Introduction

Chronic kidney disease (CKD) is a significant global public health concern, and the advanced stages significantly affect the physical and psychological well-being of both patients and their informal caregivers [1–4]. CKD is defined as the progressive and irreversible loss of kidney function for over three months, and its prevalence increases with advancing age [4]. Approximately 10% of the global population is directly impacted, and CKD is predicted to be the fifth leading cause of death by 2050 [5].

Kidney failure refers to the advanced stages of CKD, and treatment options include kidney replacement therapies such as transplantation and dialysis. However, these treatments may offer minimal survival benefits for individuals who are frail, elderly or have additional co-morbidities [1–3, 6]. An alternative option to kidney replacement therapies is conservative management (CM) [7]. Patients who opt for CM forego dialysis, with treatment focusing instead on symptom management and psychosocial and spiritual support from the healthcare team until death [7]. Interest in CM has increased in the last two decades, mainly due to increased awareness of the burden faced by older people receiving dialysis, the poor survival of patients having dialysis, and knowledge that patients receiving CM often retain a similar quality of life compared with patients on dialysis [2, 8–10].

Patients who choose CM also frequently present with multimorbidity, experience a high symptom burden, have a limited life expectancy and significant unmet palliative care needs [11]. They may feel abandoned by their healthcare team when opting not to dialyse, exacerbated by prognostic uncertainty [12]. Effective management of kidney failure is multifaceted, involving complex dietary and medication regimes, and can significantly impact those emotionally and practically involved in providing care [13]. Accordingly, informal caregivers of patients with kidney failure receiving CM may themselves have health and social care needs that must be addressed [14].

The experiences and perspectives of informal caregivers of patients with kidney failure receiving CM, particularly as the patient deteriorates towards death, are generally unknown [8]. There is a notable lack of evidence on supportive interventions for these caregivers. One reason for this is the limited knowledge about the needs and preferences surrounding psychosocial support for informal caregivers of patients with kidney failure receiving CM [15]. Most qualitative work among informal caregivers has been limited to specific treatments or situations, such as dialysis discontinuation [16–19]. In addition, the needs of informal caregivers are seldom acknowledged in policy guidance produced by the National Institute for Clinical Excellence [20] the National Service Framework [21] or the Renal Association Guidelines [22].

Therefore, it is important to explore the experiences of informal caregivers of patients receiving CM for kidney failure to identify any unmet needs and inform future supportive interventions.

### Aim

This study aims to explore the experiences of informal caregivers of people receiving CM for kidney failure, alongside healthcare professionals who provide treatment and care to people receiving CM, to identify and understand the unmet needs of informal caregivers.

## Methods

### Study design

The methodology was informed by guidance on developing complex interventions [23] and took an interpretivist approach to study design, data collection and analysis [24]. This study involved two stages. The first stage involved semi-structured interviews with informal caregivers of patients who received conservative management for kidney disease. The second stage was informed from the findings of stage 1 and consisted of focus groups with renal healthcare professionals who provided care and treatment to people with kidney failure. These data were integrated to better understand the experiences and unmet needs of informal caregivers of those receiving conservative management.

### Ethical considerations

Ethical approval was granted by the NHS Health Research Authority (HRA), Health and Care Research Wales, and REC (REC Reference Number: 22/EE/0089). The IRAS project ID was 306,036. All participants provided informed consent prior to participating in the study.

## Research setting

This study took place across two distinct UK sites, Northern Ireland (including 3 NHS trusts) England (2 NHS trusts). Participants from the sites in England were recruited from specialist CM services in large, urban hospitals serving a younger and ethnically diverse population. Participants from Northern Ireland were recruited from renal services within a range of Health and Social Care Trusts, including urban and large rural populations, with a predominantly white community and an older patient group.

### Participants

A purposive sampling technique was used to recruit 37 caregivers, 16 from Northern Ireland and 21 from England. People were eligible to participate in the semi-structured interviews if they were aged 18 years or over, had the capacity to provide informed consent, and were caring for a patient with kidney failure, who was receiving CM, and was living at home or had moved to full time residential or nursing care in the previous 6 months. Caregivers were also eligible to participate if they had been caring for a patient with kidney failure who received CM and had died in the last 2–24 months. Informal caregivers were excluded if they were caring for a patient receiving kidney replacement therapy.

For this study, we developed an operational definition of an informal caregiver as ‘A spouse, other relative, or friend, who provides support for the patient. Support / informal care may include emotional support, prompting with taking medications, observing for signs, symptoms or changes in the patient’s condition, getting prescriptions, encouraging participation in social events and physical activity, helping with household tasks, or providing physical care.’

Healthcare professionals were eligible to participate in the focus groups if they had worked for at least three months within the renal speciality and had an appropriate professional qualification and registration. They were excluded if they did not have experience delivering care to people who received CM, were employed through a recruitment agency, or did not hold a permanent healthcare contract.

### Recruitment

In stage one, informal caregivers were recruited using three approaches. First, patients receiving CM were approached by research nurses, who acted as gatekeepers and informed of the study. If the patient had an identifiable caregiver and was interested, they were given an invitation letter and information sheet to give to their informal caregiver, including a contact number, email address and self-addressed envelope if they wanted to express interest in the study. Second, some patients receiving CM may rely on informal caregivers to manage and attend appointments. Research nurses contacted named informal caregivers directly to provide them with the invitation letter and study information. Nurses followed up with caregivers by telephone after they had time to review the information sheet to confirm whether they were happy for their contact details to be passed onto the research team. Finally, the study was advertised on posters within renal clinics, on social media, and patient association websites such as Kidney Care UK and the Northern Ireland Kidney Patient Association.

Recruitment of healthcare professionals took place following the completion of the semi-structured interviews in stage 1 of the study. Stage 2 of the study was promoted over email and in regular meetings with staff at participating sites throughout Stage 1. A research team member contacted healthcare professionals at each participating site to provide an overview of the study, including a participant information sheet detailing stage 2 of the study and the contact details for the research team. The focus groups were organised at times and locations that worked best for most healthcare professionals interested in participating. An informed consent form was signed before the commencement of each focus group.

### Data collection

The semi-structured interview schedule was developed based on previous qualitative research exploring the unmet needs of caregivers of people with kidney failure [25] with additional items included to focus on end-of-life care (Supplementary Material 1). Our Patient and Public Involvement (PPI|) Expert Advisory Panel members reviewed and refined the interview schedules. A total of 38 people participated in the semi-structured interviews. Thirty-six semi-structured one-to-one interviews were conducted with 36 participants over telephone, video, or face-to-face, depending on participant preference, and one interview was conducted with two informal caregivers who preferred to be interviewed together (they provided care for the same person in their family). All interviews were conducted by one of two postdoctoral research fellows, CC and TF, who have extensive experience conducting in-depth qualitative interviews. The interviewers had no pre-existing relationship with any of the interview participants. The interviews ranged from 14 to 89 min (median = 35 min) and occurred between January 2023 and February 2024. No repeat interviews were undertaken, and interview transcripts were not returned to participants before data analysis.

The focus group interview schedule was refined following the completion of Stage 1 to ensure the exploration of the key aspects of the caregiver experience identified during the interviews (Supplementary material 2). CC and TF facilitated the focus groups. Two focus groups were conducted face-to-face, one in London and one in Northern Ireland. In addition, a one-to-one interview was conducted face-to-face in London (with a participant unable to attend the focus group), and a further focus group was conducted online.

### Data management

Interviews were audio recorded and transcribed verbatim by a third-party transcript service. Transcripts were imported into NVivo version 12 [26] and pseudonymised before coding and thematic analysis.

### Data analysis

The transcribed qualitative data from the semi-structured interviews was analysed using thematic analysis guided by King and Horrocks [27]. The first step involved descriptive coding of the data line-by-line, identifying words or phrases that captured salient components within the data. The data was coded by two researchers (TF and CC). During the second step, interpretive codes were synthesised from the descriptive codes. The interpretive codes were arranged into hierarchical categories, forming final overarching themes [28]. The process of synthesising interpretive codes was led by one researcher (CC), with the final codes being reviewed by the rest of the research team.

One researcher (AW) utilised the coding framework developed from the caregiver interviews to analyse the focus group and supplementary interview transcripts deductively. AW, CC, TF, and HN reviewed and synthesised the additional interpretive codes with the coding framework. Final themes and subthemes were revised to reflect the analysis from both groups of participants.

## Results

The majority of caregivers recruited to take part in an interview were female (*n* = 29), between 60 and 69 (*n* = 15), recruited from a London-based hospital (*n* = 22), and had been caring for their family member for between two and nine years (*n* = 17). Table 1 provides an overview of caregiver characteristics.

**Table 1:** Stage 1 caregiver interview participant demographics

Characteristic	Participants Analysed (*n* = 38)
**Gender**	
Female	29
Male	9
**Age (years)**	
20–29	2
30–39	3
40–49	6
50–59	10
60–69	15
80	1
Not reported	1
**Location**	
London	22
Northern Ireland	16
**Length of caring relationship**	
< 1 year	7
2–9 years	17
10–19 years	11
20–29 years	2
50 + years	1
**Caregiving relationship**	
*Participant is caring for*:	
Mother	17
Father	8
Mother-in-law	5
Wife	2
Husband	2
Grandmother	1
Step-mother	1
Partner	1
Uncle	1

The majority of healthcare professionals who took part in the focus group and supplementary interview were female (*n* = 14), working in a London-based hospital (*n* = 8), and with between 20 and 29 years of service (*n* = 6). Table 2 provides an overview of healthcare professional characteristics.

**Table 2:** Stage 2 HCP focus group participant demographics

Characteristic	Participants Analysed (*n* = 15)
**Gender**	
Female	14
Male	1
**Occupation**	
Consultant Nephrologist	2
Clinical Nurse Specialist	3
Sister	2
Nurse	6
Dietician	2
**Location**	
London	8
Northern Ireland	7
**Length of Service**	
0–9 years	1
10–19 years	3
20–29 years	6
30–39 years	1
40 years+	1
Not reported	3

Three themes were synthesised from the data, capturing the key facets of the caregiving experience.

### Defining the role of the caregiver

#### The daily routine of caring

Participants provided an overview of their daily caring role. Several activities focused on practical elements, including providing personal care, administering medication, cleaning the home, cooking meals, transportation to appointments, and shopping. Some activities involved high levels of organisation and time dedicated to administrative tasks.

In addition, participants monitored the health of the person they cared for by watching for changing symptoms or deterioration, ensuring that they were eating adequately, or checking blood sugar levels.

She can’t prick test herself, so basically, I check her sugar level, hoping that she maintains that. And just making sure that she’s eating the right food. She doesn’t always listen to us. But trying our best. Because obviously, if her sugar is high, that means it would affect her kidneys and just make it worse. - Caregiver 6, London.

HCPs highlighted how uncertainty around these daily activities could be a significant source of anxiety for caregivers as they navigated changing needs, resulting in worries about whether they were providing the right care.

They might worry if they’re doing the right thing. Are they giving them the right food, the right fluids, the right medications? And just, as things progress, it’s quite a big responsibility that they would be taking on. – Respondent 1, Focus group 1, Northern Ireland.

The caring role was underpinned by communication, including communication with the person cared for, healthcare services, and other family members and friends involved in providing care. For some participants, this also included relaying information between healthcare services either as a translator due to language barriers or because the patient struggled to understand what they had been told and needed reassurance. HCPs echoed this, expressing their reliance on the communication support provided by caregivers.

We rely mostly on the family members as informal carers or relatives as informal carers. Because most of these patients are very frail elderly and they have a lot of comorbidities. And considering the population that we are serving, we have a vast majority of patients whose main language is not English. So, in that way we will rely on carers to be advocates for our patients. – Respondent 4, Focus group 1, London.

Other interpersonal aspects of care included promoting social engagement by encouraging the person they cared for to contribute to conversations, get out of the house, or see their wider social circle.

While the activities were similar, daily experiences could differ depending on the health and well-being of the person being cared for. Good days were typically defined as days when the patient was well, and while this was assessed differently depending on their usual baseline health, some commonalities were seen across participants. These included engaging in social activities, getting out of bed, eating, feeling comfortable and being in a good mood.

A good day. Yes. Well, a good day would be where things are just going smoothly, where things are just going smoothly. That you get done everything that you have to get done. And she’s well, and she’s… a good day to me is when she’s eating properly, you know? - Caregiver 003, Northern Ireland.

The inverse was true in that bad days were defined by the poor health of the person being cared for, when they were unhappy, irritable, or argumentative, spent all day sleeping, were withdrawn, were not eating, or were experiencing distressing symptoms.

I think the most stressful thing lately is when dad… his personality has changed. Over the months, he’s got quite, I think, down and inward. So his sense of humour has gone and he doesn’t really talk much, and on a bad day, he would hardly speak at all. And he would sleep a lot. - Caregiver 11, Northern Ireland.

#### Changes, consequences and challenges of the caregiving role

Numerous changes came from the caregiving role, which, in turn, led to consequences and challenges that impacted the caregiver’s life, which was separate from the role itself. One common change reported, particularly if participants lacked wider support for the caring role, was a limited social life because of the time and energy needed for the caring role,

Well, it’s changed it, hasn’t it? It’s not husband and wife. We can’t go out and do things together. I am just basically a caregiver. But a caregiver who loves him, rather than just… yeah. What else can I say? We can’t do anything. He just goes out to his appointments. And we don’t have a social life. - Caregiver 1, London.

Participants also experienced changes to their living situations, with some needing to move house to provide care. They also experienced changes to their employment due to the caring time commitment and a reduced ability to take holidays.

I took early retirement six years ago, nearly seven, with the intention of starting my own business after eighteen months. But then Covid hit, and Mum and Dad came to live with us for a while, at the beginning of Covid. But then mum couldn’t cope… And at the minute, if I wasn’t doing this, looking after them, they would both be in residential care. And our aim is to keep them at home as long as we can. So I’ve just had to put my plans on the back burner for this period. - Caregiver 11, Northern Ireland.

These changes and restrictions often made participants feel they lived in a small world, as caring was constant. They could not plan for their future, leading to feelings of isolation. However, changes were not all inherently negative; the caring role often made the relationship much closer, something that participants highly valued.

Yes, it’s probably made us a bit closer. I think the last month or so. Because I had to speak to him so often, and I’ve had to talk to him about stuff, and I’ve had to sort of advise him what he can and can’t do, and make sure he has understood about things. - Caregiver 19, London.

#### Love as a motivator

Many participants described how their love for the person they cared for motivated them to take on the caring role and ensured that it felt inherently rewarding. Participants acting as caregivers for a parent felt the caring role was their way of ‘giving back’ for the care they had received as a child. Often framed through a lens of obligation, it was not viewed negatively but instead instilled in participants a sense of duty they felt honoured to fulfil.

When I give my mum a bath, it’s like I’m giving my daughter a bath. She’s just like a child now. And it’s just so beautiful that I can serve my mum. Because, like I said, when we were growing up, we didn’t think of Mum at all. Mum was just mum. So now is the time that we can serve her, just like she served us when we didn’t know any better. So, it’s a very beautiful cycle because you appreciate what your mum did - Caregiver 8, London.

Caring was seen as an opportunity for participants to spend more precious time with the person they cared for. This provided a positive perspective on potentially distressing situations, for example, their experience of the COVID-19 pandemic or the patient’s diagnosis of an end-stage illness. This love they felt towards the person they cared for and the appreciation of the time spent together ensured that the caring role was viewed as an inherently rewarding part of their lives. Some carers took pride in being best placed to provide the highest quality care to the patient due to their closeness. This was reflected in the perspectives of carers who had already experienced a bereavement and who expressed reassurance and comfort, knowing they could provide care to the person they loved when it was needed.

And at least, now that he’s not here, you tried to make the best … made it as best for him as you could. And that’s comforting, you know. - Caregiver 5, Northern Ireland.

For some participants, this translated into the caring role having a profoundly positive impact on their well-being and perspective on life.

I told myself that I’m not impacting my life. If anything, I am making it better, because I am putting myself through an experience where it has made me a better person. It has given me a better understanding. Strengthened my morals and my understanding in life. …. And it just made me open my eyes, thinking life really is very short. Let’s make the most of it. Do as much good as you can. - Caregiver 10, London.

However, HCPs reflected on how the loving relationship they observed between caregiver and patient could result in a paternalistic dynamic, where carers desire to shield the patient from bad news. This was highlighted as creating challenges when communicating with patients, particularly when language barriers required the caregiver to relay information about the condition and treatment to the patient.

They don’t want to disclose the patient that she is not for dialysis. Because it’s going to break their heart, you know emotion, they will be constantly very unwell, low mood and they will not want to take their medication if you are doing, and they think they might die very soon. So the family don’t want to even disclose the information to the patient. - Respondent 1, Focus group 2, London.

### Keeping the end in Mind

#### Awareness and attitudes towards death

Most carers described an acute awareness of death as the patient’s disease progressed. Some had previously experienced the death of family members or friends, requiring end-of-life care. Participants’ attitudes towards death were coloured by past experiences, meaning that if they had negative experiences, they felt less comfortable with discussions around death. In contrast, positive experiences were found to be reassuring and comforting.

To be honest with you, I don’t like thinking about it. I just sort of take it as it comes. Last year, we lost my father-in-law, and it was very unexpected. And she was ill before him anyway, and he just suddenly fell ill. And I think having to deal with that, it’s sort of now… even before it, I suppose, we don’t really sit and think, oh my God, what are we to expect? What’s coming?. - Caregiver 13, London.

However, there were distinct differences in attitudes towards death among carers in London and Northern Ireland. Participants in Northern Ireland were more reluctant to discuss death and dying with their loved ones or to confront the reality that the person they cared for had an end-stage illness.

She’s never thought of that either. She’s such a fighter and she just gets on with it. As I explained earlier on, she has so many things, so many health issues. But to sort of put this into a box, I don’t think for us it would be helpful. Put it that way. - Caregiver 8, Northern Ireland.

This contrasted with carers in London, who were more likely to have strong religious beliefs that informed their approach and awareness of death.

Life is such that we are temporary guests in this world. We came, and we will go back. So, it’s just part of life. And then, whatever time you have, you appreciate it, you take advantage of it and you make the most of it. And then, when we all die, we will be together in heaven. So, you feel sad, but at the same time, it’s not the end of the world. We will all go, but we will all be in our eternal life where there will be no one there. It will just be happy, contentment, pure bliss. - Caregiver 8, London.

For these carers, this allowed them to frame death in a positive light, recognising that there was a possibility to have a ‘good death’ and made them appreciate the time that they had with the person they were caring for. HCPs also reflected on the differing attitudes to death observed across different cultures.

I think that that’s a more … western society; we are very much more, as I said, more individualistic, and we are more like the needs of the individual person, the individual patient. We are very much about patient rights and, respecting those rights and making sure that people are informed. And that’s just not the same, necessarily, in a significant part of the world population. So I’m always having to remind myself of that. - Respondent 4, Focus group 1, London.

Carers described how being able to discuss death openly with HCPs could be reassuring and comforting.

Well, the doctor whom we saw face-to-face said,… and this is the only thing that we’ve really been told about the progression… he said, it’s not a bad way to go. You sort of become more and more sleepy. And so, this is it, really. You fade away, in a way. So, it sounded not too painful. - Caregiver 21, London.

#### Unknowns at the end of life

There were many questions and uncertainties about disease progression and end-of-life. Some questions were impossible to answer with certainty, such as those about life expectancy. HCPs reported that carers often wanted to know when the patient might die, and a key challenge of their role was managing and supporting carers in this uncertainty,

It’s difficult to say to someone precisely when things are going to change, because everyone is very different. And … and there is explanation regarding, … how patients very often live longer without dialysis than with dialysis. So it’s trying to help them understand all of that. That by making the decision not to have dialysis doesn’t necessarily mean that you’re going to die sooner. - Respondent 7, Focus group 1, Northern Ireland.

This inability to have definitive answers about timelines of disease progression was identified as one of the most difficult aspects of the role by carers,

But I think the thing we find most difficult is not knowing how long she’s got left. -Carer 13, Northern Ireland.

Other questions carers had were more pragmatic and focused on decision-making at the end of life. For example, when it would be appropriate to call an ambulance, what would the care needs be, and what resources would be available. However, HCPs described how these questions linked to more complex concerns and issues, such as managing family dynamics during decision-making processes and managing culturally sensitive conversations around end-of-life care and advance care planning.

Some family members were aware of the care plan, but then other family members… no, I’m the eldest in the family, I should be respected. And this is my decision. And the family member actually knows the patient’s wishes. Are actually crying, oh please, please, Dad doesn’t want to do that. … so mostly, we have Asian population and … there’s a decision maker in the family, so the eldest is decision maker. I’m the eldest. This is my decision. Even if the youngest was saying, but Dad doesn’t want it. No, I’m the decision-maker. He should go to hospital. So it’s tricky. - Respondent 4, Focus group 1, London.

Overall, carers reported a lack of knowledge about the end of life. This was not necessarily because the information had never been provided but because the timing of the information meant it was easily forgotten, dismissed, or felt less relevant at that time.

When she was initially diagnosed, we were given this booklet. So maybe I should have a read through that. At the time I did, I was just… yeah, just went over my head, but I didn’t read it at the time. - Caregiver 2, London.

HCPs described the difficulties of managing the timing of information delivery. They felt caregivers may require consistent, repeated education and reassurance, even if they received the relevant information earlier in the patient’s journey.

Once the patient becomes unwell, that is the time that they ask you so many questions. But they only care once the relative is symptomatic. When someone is dying, that’s the only thing, oh you didn’t tell me at the time… they bombard you., why you didn’t tell me this? Even if you tell them this information was given that time, but still they still deny, even though you document. - Respondent 1, Focus group 2, London.

Despite questions and uncertainties, some carers did not want to think about disease progression or dwell on what would happen in the future and instead manage the ‘here and now.’

If something happens, then I’ll cope with it then. I’ll deal with it. I’m good at organising things. I’m very practical, so I can organise things as needs be. - Caregiver 8, Northern Ireland.

However, this approach did not necessitate ignorance. Instead, it demonstrated an important coping strategy of remaining in the present moment when providing care, which could be facilitated by providing information. A small number of carers had professional knowledge or previous personal experiences of death. As a result, these carers were able to live in the present without a high level of anxiety about the future unknowns.

If the kidneys start to fail, obviously managing all of that sort of end-of-life care and all of that. But I think because I have been through it with [name], and even though [name] was in a home, I kind of am fully aware of what lies ahead in that area. But I don’t think too much about it until I have to. Because I don’t believe in panicking too much, you know? - Caregiver 12, Northern Ireland.

#### Influences on decision-making

The experience of choosing CM differed depending on the patient’s health, the size and involvement of the wider family, their culture, and the caregiver’s understanding of kidney failure and dialysis. Most caregivers had some degree of involvement in the decision-making process. Some caregivers and HCPs described how the family was divided over the decision.

I think my sisters took that… definitely, the middle one took that quite bad. Why does he not want to be resuscitated? I’m like, he doesn’t. I didn’t bat an eyelid. That’s his decision. But she was probably trying to convince him out of it. People have to make the choices that they want to. - Caregiver 5, London.

These divisions could strain the relationship between carers, patients and HCPs. One participant described how they did not trust the decision that the person they cared for had made and felt HCPs had not managed the decision-making process correctly.

Initially, I was in a situation where I know she’s saying no, but I know my mother that when the time comes, she’s going to be like, I want it done, I want it done. So you’ve got to change the approach with her. But the hospital and the doctors felt that, oh no, it’s you who want her more to go on it than her. We have to respect her wishes. - Caregiver 16, London.

These divisions between patients and carers in decision-making were particularly problematic at the end of life when family caregivers who were not prepared for the worsening of symptoms might overlook patients’ wishes.

There are chances where, even if they have that [universal care plan], patients still go to hospital because the family member themselves will take them to hospital. So there are some patients wherein they are actually dead on arrival to A&E. - Respondent 4, Focus group 1, London.

In contrast, most carers appreciated the involvement of HCPs. HCPs either helped reassure carers that the decision that had been made was the best one from a clinical perspective, or at times, it was HCPs themselves who made the decision on behalf of the patient, typically because of the limited benefit of dialysis.

I think initially we were like… because as a family, they all sat down… but she was adamant. And when the doctor … I think because the doctor also said it’s not a good idea, and I think that kind of reassured them that maybe… you know, we’ll just follow her. - Caregiver 6, London.

The main priority that underpinned the decision-making process was assessing quality of life. This assessment was driven by factors such as travelling for dialysis, fears of pain and discomfort, previous experience of undergoing a high treatment burden for other conditions, or observing friends or relatives who had undergone dialysis. Understanding the reality of dialysis was key to facilitating certainty in the decision to opt for CM.

She said all along that there was no way she wanted to have the dialysis. And so whatever the team that worked on her worked around knowing the fact that she never really wanted dialysis. So, touch wood, in that respect, everything has worked out OK, I suppose. - Caregiver 11, London.

Risk was also considered during the decision-making process, alongside the potential benefit of added years of life. Some participants understood that there was limited survival benefit to dialysis due to the age or frailty of the patient. In contrast, others explained that the presence of cardiovascular co-morbidities meant dialysis was not appropriate. As a result, most carers were comfortable with the decision due to this understanding of priority and risk. In one case where a carer was unhappy with the decision to choose CM and not receive dialysis, this appeared to reflect a misunderstanding of how age and frailty influence the risks and benefits of dialysis.

If you look at the situation she is in, she’s quite stable. So that’s why it was easier for her to use that easy option out, that I don’t want to get dialysis done. But now that we’ve sat down and explained to her, and said look, there’s hundreds of thousands of people are doing this every day, twice a week, three times a week or whatever. And it’s not going to be easy for the family, but it has to be done. And we are ready for it. And she’s got to understand that and slowly, slowly. So now she’s come around that, and she’s said, OK, I want to live still. - Caregiver 20, London.

### Balancing the burden

#### Caregivers’ coping strategies

Carers described a range of coping strategies used to manage the difficulties of the caregiving role, including religious faith. However, the specific religious beliefs differed between carers in London and Northern Ireland. In London, most carers who had a strong religious belief were Muslim, and their faith provided a strong sense of altruism, duty, and gratitude for having the opportunity to serve their loved ones and their faith within the caring role.

And obviously because of our religion, I am so grateful to God that he has given me the opportunity to serve my mum. Seriously. Obviously, it has an effect on me as a mum myself. And obviously some days … I appreciate the impact. I think it has made me more conscious of who I am and my purpose in life and what I should be doing. - Caregiver 8, London.

In contrast, most in Northern Ireland who reported strong religious faith were Christian. Carers described how their Christian faith provided reassurance as they believed they knew what would happen following death.

We are a Christian family, we are all saved, you know? And when daddy died, that’s the one thing that gave us all comfort, was knowing where he was. And it will be the same with mummy. […] If she didn’t have her faith and we didn’t have ours… but as I say, when that time comes, we will just… we will cope with it. - Caregiver 4, Northern Ireland.

Participants described how taking time to themselves, away from their caring role or other obligations, was also important to ensuring they were able to cope with the demands of caring. Taking time to themselves provided participants with a distraction and an opportunity to nurture parts of their lives that were not connected to their caring role, for example, by taking up hobbies or activities such as yoga, meditation, listening to music, watching TV, or going for a walk.

I like to just have some quiet time, so it might just be listening to music, watching music documentaries on TV, watching Strictly Come Dancing at the moment! And also, I find that what works for me is to really structure my routines, my week. So, I know that on most Wednesdays I go out with one of my friends for dinner. So, it’s just organising and managing my time, I think. - Caregiver 18, London.

#### Facilitators that ease the caregiving burden

Separate from coping strategies, caregivers described factors that helped reduce the impact of caring by reducing the overall burden. Broader family support for caring provided flexibility to the role when others contributed to caring, reducing the demands on each individual person. For participants who did not have broader family support or where the caring demands exceeded what the family could provide, the provision of paid caregivers was an essential facilitator. Additional support from family or paid caregivers could allow carers to continue working or going on holiday while maintaining their carer role.

So I have now put in place… because I need my freedom, I need a bit of respite as well… so I have now put in place… and this is private… I’ve got two caregivers, two lovely ladies now, who I’ve got to know. And they are … I’d like to get away every few weeks, two nights away. - Caregiver 1, London.

HCPs also recognised that sharing the caregiver role across multiple people, with support from different agencies, was essential for managing the high burden caregivers faced. When caregivers lacked additional family or paid support, improved access to social services and financial resources was needed due to the impact on the carer’s ability to maintain employment.

They do get worn out because they don’t have any other support from the family. They are the only one that’s looking after that patient themselves. And I think sometimes they have personal family matters as well, going on at the same time. …some of them, they don’t have family support at all, or they don’t have any friends. Just the two of them; the patient and the carer. - Respondent 1, Focus group 2, London.

Separate to external support, a key factor facilitating the caring role was an optimistic outlook, and an ability to be positive in profoundly difficult situations. Carers described being able to shift their perspective to find positives in different situations.

There’s nothing about this that we can change. So if things are sliding along and they’re pretty tickety boo, maybe by somebody else’s standards they are not tickety boo, but from where we are, at this point in her life, she’s just sailing along. - Caregiver 3, Northern Ireland.

#### Healthcare services

Carers had extensive experience navigating the healthcare service particularly as most patients had multiple conditions. These experiences could reduce or increase caregiver burden. This hinged on accessibility and communication within and between services. The experience within renal services specifically reduced the overall burden on participants. Participants described consistent communication, good rapport, easy access and consistent, reliable support from the CM team. In particular, knowing who to contact when they had questions was particularly effective at reducing burden.

Well, we are having these calls now, every six months, and then any questions I do get… What else would I need? No, I can’t really think. I can’t think of anything else that I would need. No, I can’t think. I know [name] is on the end of a phone, or she gets back to me the next day or whatever. So that’s comforting to know. - Caregiver 1, London.

HCPs highlighted the impactful relationship between the supportive care nurses and carers, and recognised that they had become the main resource for reassurance, guidance and emotional support. However, this high level of support was identified as potentially burdensome and unsustainable for the nursing staff

We do a lot of emotional support to them as well, and it’s quite taxing on our part. And sometimes, I would just go out of the room and just take a deep breath. Especially during the day, you have a lot of calls regarding providing emotional support to them… we did have clinical supervision before, but it’s no longer happening. Clinical supervision did help us with regards to being overwhelmed with emotional problems that our carers are presenting to us. - Respondent 4, Focus group 1, London.

There were far more mixed experiences of primary care services. Negative experiences compounding caregiver burden included poor continuity of care and understanding about kidney failure and CM, and delays in follow-ups and referrals. These issues resulted in time spent navigating the service, advocating for referrals or further tests, and consumed significant amounts of time and energy. Difficulty accessing services and lack of continuity of care and communication were common across multiple healthcare services. Carers described their loved one falling through the cracks while understanding that these issues resulted from a lack of resources and growing waiting lists in the NHS. These difficulties were particularly amplified when patients had complex needs due to multi-morbidity. These issues were also experienced by HCPs, who expressed difficulties in coordinating multidisciplinary services and ensuring continuity of care for patients under conservative management.

A lot of patients who are being conservatively managed are attending a lot of different clinics for a lot of different things. And their other comorbidities may well predict their quantity and quality of life much more than their kidney failure. But I think that when people are under a nephrology clinic of any sort, it does seem to be a banner headline on their problem list. And I think other specialities are anxious to make pragmatic decisions about escalation. - Respondent 3, Focus group 1, London.

Carers were also not provided support through healthcare services for their wellbeing and felt the healthcare services were focused on treating the patient alone. This issue was highlighted by HCPs who felt carers received adequate follow-up support following the diagnosis of end-stage illness,

When you look at the mortality and morbidity associated with chronic kidney disease, and what we’re running in low clearance is not dissimilar to a cancer… we do not have anywhere near the support for giving bad news here. - Respondent 5, Focus group 1, London.

## Discussion

This study qualitatively explored the experiences of informal caregivers of people receiving CM for kidney failure, alongside healthcare professionals who provide treatment and care to people receiving CM, to identify and understand any unmet needs.

Caregivers described changes from taking on the caregiving role and how their lives were centred around their daily caring tasks. This is similar to the experiences of carers of people with advanced cancer, who can experience a shift in their identity as they struggle to detach their own sense of self from the caring role [29]. This was further emphasised in our study by carers who defined their experience exclusively within the context of the person that they care for. For example, defining their categorisation of good and bad days solely on the health and wellbeing of the person they care for, as opposed to other aspects of their life, work or relationships. In some participants caring for a parent, this extended to a profound change in identity and relationship dynamic with their parent, as they experienced a reversal of roles and identity through parentification [30].

However, unlike other qualitative research into the experiences of caregivers of those with advanced end-stage illness [30] we found that changes in daily life were multidimensional and not inherently negative. Parentification was predominantly perceived by caregivers within the context of reciprocity and filial piety [31] particularly among participants with strong religious beliefs. Culture can influence motivations for undertaking caregiving and the perspective of the caregiving role, with some caregivers viewing the role as a source of pride [32]. However, cultural expectations can also influence the expectations around caregiving, with most of the responsibility falling to women within a family [33]. Unsurprisingly, most of the participants in our study were women, although both male and female caregivers expressed a sense of obligation and filial piety. Cultural and familial expectations can also influence conflict within family units during any decision-making processes and in providing care [34] which caregivers and HCPs highlighted in our study for participants with large, close family units. Despite this, sharing the caregiving responsibilities across family members was identified as a facilitator for the caring role and can ease the caregiving burden by reducing the time each individual dedicates to caregiving tasks and loneliness [35].

The process of death and dying was unknown for most caregivers and something that healthcare professionals could not adequately predict. While caregivers reported receiving information about disease progression during the decision-making process for CM, they could no longer recall the details. However, the questions that caregivers had were not necessarily easy to answer, as they predominantly focused on the anticipated care needs of the person they cared for and how much time the person they were caring for had left to live. These questions are in line with caregivers’ desire to be in control and feel prepared for deterioration and the dying process [36], despite the difficulty that HCPs have in answering those questions with certainty on an individual level [37].

Other than the desire for more information about disease progression, there were few consistently reported unmet needs. We found that the experience of providing care was multidimensional and not inherently negative. Previous research has highlighted that caregivers of those who have chosen CM experience better care-related QoL than those who choose dialysis. This difference could be related to the knock-on impact of high treatment and symptom burden on the wider familial and social circle [38]. Additionally, when providing care for people at the end of life, the burden can be viewed as more manageable to some because there is an awareness that the caring role is temporary and will only last for a limited time [38].

Overall, the key aspect of the caregiving experience that helped caregivers cope with their role was support from the renal team and effective communication. Caregivers described the benefit of having someone to go to directly with questions and the difficulties they experienced with other services when communication was poor. Previous research into the experiences of palliative care emphasises the importance of communication with patients and caregivers, as well as across services, to ensure people feel adequately supported [39, 40]. However, this can be time- and resource-intensive, significantly adding to the burden of HCPs within the renal team. This highlights the importance of adequate resourcing and staffing within these services to ensure that HCPs are not at risk of burnout [41].

### Strengths and limitations

This qualitative study was conducted across multiple sites in the UK with substantially different demographic characteristics (Northern Ireland and London). We were able to collect data from a range of carers with different caring relationships and cultural backgrounds, and we were also able to conduct focus groups and interviews with healthcare professionals to triangulate and expand on our findings from stage 1. However, as this is a qualitative study and only includes the experiences of caregivers and healthcare professionals from five sites, the findings may not be transferable to renal units across or outside the UK. Additionally, we only collected data from caregivers at one point. We did not follow up caregivers to explore how the experiences changed as the person they cared for approached the end of life. We also did not capture healthcare professionals’ experiences in other services involved in delivering care, such as primary care, hospice or palliative care services. We did, however, include caregivers who had been bereaved and were able to recount their experience throughout disease progression to ensure that aspect of the experience was not missed.

## Conclusion

In conclusion, we found that informal caregivers of people with kidney failure who chose CM had multifaceted experiences in their role and felt well-supported by renal healthcare teams overall. Cultural factors, such as religion and family expectations, were found to strongly influence caregivers’ experiences of the role in a mostly positive way. However, this could lead to tensions during the decision-making process. Communication and the provision of information were identified as the key needs of caregivers. While this was mostly addressed within existing services among the caregivers in this study, renal healthcare services must be well-resourced to ensure that high-quality care can continue.

## Electronic supplementary material

Below is the link to the electronic supplementary material.

Supplementary Material 1

Supplementary Material 2

## Data Availability

The data that support the findings of this study are available from the authors on request. As the data is qualitative the raw data contains potentially identifiable information, therefore restrictions apply to the availability of these data to maintain confidentiality. The data set for this study can be provided by contacting the chief investigator, Professor Helen Noble, at helen.noble@qub.ac.uk.

## Abbreviations

CKD: Chronic Kidney Disease
CM: Conservative Management
NHS: National Health Service
UK: United Kingdom
