# Supplementary material for: A qualitative exploration of cAregiver experienCes Of conseRvatively maNaged kidney failure: the ACORN study
Source: BMC Nephrol. 2025 Jul 1;26:303. doi: 10.1186/s12882-025-04209-w (PMC12211965; doi:10.1186/s12882-025-04209-w)
Supplement: Supplementary file 1 — Supplementary Material 1 [file 12882_2025_4209_MOESM1_ESM.docx]

# **ACORN Study**

# **Stage One: Participant Interview Schedule**

The purpose of this interview is to explore the experiences and unmet needs of informal caregivers of people with end-stage kidney disease who chose not to undergo dialysis.

**General experiences associated with caregiving.**

**Question:** Tell me about the person who you care for. Tell me about your caring role.

**Prompts:** What is their name, age, and when were they diagnosed with ESKD? What specific tasks do you associate with your caregiving role, for example personal care needs, medications, food and fluid requirements, monitoring loved one’s well-being, liaising with healthcare professionals, managing hospital appointments

**Question:** Tell me about a typical ‘good day’ of providing care for [insert name].

**Prompts:** Can you tell me about some positive experiences associated with your caring role? What do you find most rewarding about providing care?

**Question:** Tell me about a typical ‘bad day’ of providing care for [insert name].

**Prompts:** Can you tell me about some negative experiences associated with your caring role? What do you find most distressing about providing care? What challenges or difficulties might you encounter when carrying out your caring duties?

**Question:** In what ways has providing care for [insert name] influenced your relationship with them?

**Prompts:** What positive / negative impacts has the caring role had on your relationship?

**Question:** In what other ways has providing care for [insert name] impacted your life?

**Prompts:** In what ways has providing care impacted other relationships with friends and family, your social life, your working life, etc.

**Experiences of ESKD, conservative management and decision-making**

**Question:** Tell me how your loved one responded to being diagnosed with ESKD.

**Prompts:** Does the patient talk about their condition, how did they cope with being diagnosed?

**Question:** Tell me how you responded to your loved one being diagnosed with ESKD.

**Prompts:** How did you find out that your loved one had ESKD, tell me about the support that you received from the renal service, do you talk about your loved one’s condition with them?

**Question:** Tell me about how your loved one started conservative management/supportive care [*depending on the terminology used by the service*]. How did they respond to starting conservative management/supportive car? How did you respond?

**Prompts:** Were you involved in the decision for your loved one to start conservative management/supportive management/ to not receive dialysis, in what way? Tell me about how this decision was made. What was the most important thing that contributed to the decision?

**Question:** Thinking back to when your loved one started conservative management/supportive management, what would you have found useful or would have been of benefit to you at this time?

**Prompts:** From experience, what would help future carers, assist them in their caring role or help them cope with coming to terms with ESKD.

**Current support and coping strategies**

**Question:** Can you tell me about the type of support/assistance you receive?

**Prompts:** What type of informal and formal support do you receive? Where do you typically seek support? What type of healthcare professionals provide you with support?

**Question:** Tell me about the ways you receive informal support yourself, for example from friends and family.

**Prompts:** Who provides you with support? In what way do you friends and family help support you in your caring role, what role does social support play in your mental health and wellbeing?

**Question:** Tell me about the ways healthcare professionals support you in your caring role.

**Prompts:** Do you know where to access support? Where do you receive support from healthcare professionals, what type of support do you receive from healthcare professionals? Has the support you received changed over time? In what way? Tell me about a time when you received support from a healthcare professional.

**Question:** Tell me about ways you feel the professional support that you receive could be improved.

**Prompts:** What support do you feel you need as a carer? Is there anything missing from the support you receive/ have received in the past, is there anything you would like more support with? Are there any barriers you have experienced when trying to access support?

**Interviewer:** How do you cope with the challenges and difficulties associated with your caregiving role?

**Prompts:** Tell me about an example of an instance when you took time for yourself. How did this help, what did you do? What do you do to relax and take care of yourself?

**Disease progression and end-of-life**

**Question:** When you think about your loved one’s ESKD and what lies ahead for them and for you (say, the next few months or year) what comes to mind for you?

**Prompts:** What are the concerns and hopes that you have for the next coming months? What information have you received about what to expect over the next few months or years.

**Interviewer:** Thinking about the future, tell us what support you think would be of most use to you as (insert name)’s disease progresses.

**Prompts:** Do you feel you will need more support or different types of support in the future – if so, in what way? Do you currently have any questions about the future that haven’t been answered?

**Question:** What information do you feel would help you plan for the future caregiving needs of [insert name]?

**Prompts:** Do you currently have any questions about the future that haven’t been answered? What concerns do you have about your ability to provide care for [insert name]

**Closing the interview**

**Interview:** Is there anything else you would like to say about your caregiving role that you haven’t had the chance to tell me?
