# Supplementary material for: A qualitative exploration of cAregiver experienCes Of conseRvatively maNaged kidney failure: the ACORN study
Source: BMC Nephrol. 2025 Jul 1;26:303. doi: 10.1186/s12882-025-04209-w (PMC12211965; doi:10.1186/s12882-025-04209-w)
Supplement: Supplementary file 2 — Supplementary Material 2 [file 12882_2025_4209_MOESM2_ESM.docx]

# **ACORN Study**

# **Stage Two: Focus Group Interview Schedule**

The purpose of this focus group is to explore the experiences and unmet needs of informal caregivers of people with end-stage kidney disease do not start dialysis, an approach to care known as conservative management.

**General experiences associated with caregiving.**

**Question:** Tell me about your role and your experience of providing healthcare to people who have end-stage kidney disease and are receiving conservative management.

**Prompts:** What does a typical day look like in your job? What support do you provide in your role to people who are receiving conservative management?

**Question:** Tell me about your understanding of the role of informal caregivers in the care of people receiving conservative management?

**Question:** Tell me about how you involve informal caregivers in your own practice.

**Prompts:** Can you tell me about an experience of working with informal caregivers to provide care to a patient who is receiving conservative management.

**Question:** From your experience working with the informal caregivers, what do you feel are the biggest challenges of the caring role?

**Question:** Many patients who choose conservative management have multiple chronic conditions. How do you feel informal caregivers manage these complex health needs of their loved ones?

**Prompts:** What support do you provide carers to help navigate complex health needs? Are there any aspects of multimorbidity that you feel are especially difficult for carers to manage, what are these?

**Question:** Can you tell me how your service works alongside other services, including primary and secondary care, to deliver conservative management.

**Prompts:** Are there any challenges to working across multiple services. Can you tell me about an experience when cross-service working was challenging? Can you give me an example of when cross-service working was effective and led to good outcomes?

**Experiences of ESKD, conservative management and decision-making**

**Question:** Tell me about the typical timeline of kidney failure diagnosis and how decision making is supported in renal services when conservative management/supportive care [*depending on the terminology used by the service*] is being considered.

**Prompt:** Tell me how conservative management is explained to, and offered to, patients within your service. What do the conversations around conservative management/supportive care [*depending on the terminology used by the service*] typically look like?

**Question:** Tell me about how informal caregivers, such as family and friends, are involved in this process.**Prompt:** What do you feel is the most difficult part of this process for informal caregivers?

**Question:** What are the typical questions that informal caregivers have about conservative management during this process.

**Prompts:** What are the most common concerns that caregivers raise when discussing the decision for conservative management

**Question:** What information is provided to informal caregivers during the decision making process

**Prompt:** How do you feel caregivers respond when receiving this information? How is information is provided to informal caregivers during the decision-making process? What improvements could be made to help informal caregivers understand the decision and prepare for their role in conservative management.

**Current support and coping strategies**

**Question:** Can you tell me about the type of support/assistance you provide to informal carers once a patient has started when conservative management/supportive care [*depending on the terminology used by the service*]?

**Question:** Can you tell me about your experience of the outlook of informal caregivers during the conservative management process

**Prompts:** From your experience how do informal caregivers react to the progression of disease, is it with hope, despair, fear etc., and how do you manage this in your professional role?

**Question:** What are the gaps in support for informal caregivers and how do you feel support for informal caregivers could be improved?

**Prompts:** Are informal caregivers well-supported in terms of information, psychological and social support, financial or practical support? If not, where are the gaps in support? If yes, what support is particularly helpful?

**Disease progression and end-of-life**

**Question:** Can you tell me about your involvement in the care of patients who are progressing towards the end of life.

**Prompts:** What does the typical care pathway look like for someone receiving conservative management.

**Question:** Can you tell me about what and how information is provided to the informal caregiver about disease progression and end-of-life.

**Prompts:** How is this information communicated and when is it communicated? How could this process be improved to ensure informal caregivers feel more prepared for the end of life.

**Question:** What support is currently available within your trust for informal caregivers at the end of life? What support do you feel is still needed?

**Question:** From your experience, what are the most difficult experiences and challenges that informal caregivers encounter as end-of-life approaches

**Prompts:** Do you feel informal caregivers are prepared for disease progression and end-of-life? If not, why? If yes, what do you feel is helpful for informal caregivers to ensure they are prepared. Tell me about a time when you provided support to an informal caregiver towards the end of life.

**Closing the focus group**

**Interview:** Is there anything else anyone would like to say that they haven’t had the chance to tell me?
